# Spinal Muscular Atrophy, types I and II: What are the differences in body composition and resting energy expenditure?

**DOI:** 10.1016/j.clnu.2016.10.020

**Published:** 2017-12

**Authors:** Simona Bertoli, Ramona De Amicis, Chiara Mastella, Giulia Pieri, Ester Giaquinto, Alberto Battezzati, Alessandro Leone, Giovanni Baranello

**Affiliations:** a International Center for the Assessment of Nutritional Status (ICANS), Department of Food Environmental and Nutritional Sciences (DeFENS), University of Milan, Milan, Italy; b S.A.PRE., Early Habilitation Service, Mangiagalli e Regina Elena Hospital, Milan, Italy; c Dietetic and Nutrition Center, M. Bufalini Hospital, Cesena, Italy; d Developmental Neurology Unit, Carlo Besta Neurological Institute Foundation, Milan, Italy

**Keywords:** Spinal Muscular Atrophy type I, Spinal Muscular Atrophy type II, Body composition, Body water, Resting energy expenditure, Nutritional status

## Abstract

**Background & aims:**

Different neuromuscular functional domains in types I and II Spinal Muscular Atrophy (SMAI and SMAII) could lead to differences in body composition (BC) and resting energy expenditure (REE). Their identification could provide the key to defining appropriate strategies in clinical dietary management, but data comparing SMAI and SMAII in terms of BC and REE are not yet available. We measured total and regional fat (FM), lean (LBM), mineral (BMC) masses, body water (total, intra- and extra-cellular, TBW, ICW, ECW) and REE in a sample of SMAI and II children, matched for age and sex, and also adjusting for body size to compare these features of the two SMA phenotypes.

**Methods:**

15 SMAI and 15 SMAII children, (M/F = 9/6 vs 9/6, age 3.6 ± 1.9 vs 3.5 ± 1.8 years, p = 0.99), confirmed genetically, were measured as follows: Anthropometric measurements [Body Weight (BW), Supine Length (SL), Arm Length (AL), Femur Length (FL), Tibia Length (TL)], Dual x-ray Energy Absorptiometry (DEXA) [total and segmental FM, LBM, FFM, and BMC], Bioelectrical impedance (BIA) [TBW, ICW, ECW] and Indirect Calorimetry (REE, respiratory quotients) were collected by the same trained dietician. BW, SL and Body Mass Index (BMI) Z-scores were calculated according to CDC Growth Charts (2000).

**Results:**

SMA children had high percentages of FM and a lower percentage of TBW and ECW compared to the respective reference values for sex and age, whereas the BMC percentages did not differ, even splitting the two phenotypes. SMA I children had a lower BW and BMI-Z score compared to children with SMA II, but similar total and segmental FM. On the contrary, total FFM and LBM were significantly lower in SMAI (7290.0 ± 1729.1 g vs 8410.1 ± 1508.4 g; 6971.8 ± 1637.1 g vs 8041.7 ± 1427.7 g, p = 0.039, p = 0.037, respectively), particularly at the trunk level. Arm BMC also resulted significantly lower in SMAI. The measured REE values were similar (684 ± 143 kcal/day vs 703 ± 122 Kcal/day p = 0.707) whereas REE per FFM unit was higher in SMA I children than in SMA II (95 ± 12 kcal/FFMkg vs 84 ± 11 kcal/FFMkg p = 0.017).

**Conclusions:**

This study has shown that BW and BMI Z-score measurements alone can be misleading in assessing nutritional status, particularly in SMAI. The differences between SMAI and II in total and regional BC are related only to FFM, LBM and BMC, and seem to be more linked to the magnitude of neurofunctional impairment rather than to the nutritional status derangement. SMA I and SMA II children can have different energy requirements in relation to their specific BC and hypermetabolism of FFM. Based on these results, our recommendation is to use direct BC and REE measurements in the nutritional care process until SMA-specific predictive equations become available.

## Abbreviations

AL: arm length
BC: body composition
BIA: bioelectrical impedance
BMC: bone mineral content
BMD: bone mineral density
BMI: body mass index
BW: body weight
DEXA: dual X-ray energy absorptiometry
ECW: extra cellular water
FFM: fat free mass
FFMI: fat free mass index
FL: femur length
FM: fat mass
FMI: fat mass index
ICW: intra cellular water
LBM: lean body mass
REE: resting energy expenditure
SL: supine length
SMA: Spinal Muscular Atrophy
SMAI: Type I Spinal Muscular Atrophy
SMAII: Type II Spinal Muscular Atrophy
TBW: total body water
TL: tibia length

## 1 Introduction

Spinal Muscular Atrophy (SMA) is a rare (1:6000–10,000 live births) [1], [2] autosomal recessive neurodegenerative disease characterized by degeneration of spinal cord motor neurons, atrophy of skeletal muscles, and generalized weakness [3]. The classical form is due to deletion, conversion, or mutation of the survival motor neuron 1 (SMN1) gene [4], and it is clinically categorized into four phenotypes according to onset age and clinical progression [5]. The most common and severest forms are SMA type I (SMAI) and type II (SMAII) [6]. Babies with SMAI show hypotonia and muscle weakness, at birth or within a few months of life, never acquiring a sitting position [7]. They have difficulty in breathing and swallowing [8], and require early mechanical ventilation and artificial feeding, which has a significantly impact on their overall health. Instead, the onset of SMAII is characteristically before 2 years of age (7–18 months) [7], and although the affected children can sit without support they are never able to walk independently. Once again also the SMAII form has a quite common condition of “weak” swallowing [9], together with chewing and respiratory problems, leading to mechanical ventilation [7]. However the nutritional status of the two forms has also been reported to be quite the opposite: SMAI children are inclined to be underweight [10] while SMAII children can be at risk of being overweight or even obese [11], in a percentage of cases malnourishment is also reported [9], [11], [12].

The different neuromuscular functional domains and nutritional status profiles could lead to differences body composition (BC) and resting energy expenditure (REE) which is highly affected by BC. However, data comparing SMAI and SMAII in terms of BC and energy metabolism are still not available.

Dual X-ray energy absorptiometry (DEXA) was apply to study BC in SMAI and SMAII children compared with healthy peers, showing total body fat mass (FM) increase and total body fat free mass (FFM) and bone mineral density (BMD) decrease [10], [12], [13]. Unlike FM, FFM is a multicomponent compartment including, at a molecular level, water (73%), protein (19%), mineral (8%, including osseous and non-osseous mineral) and glycogen (0.1%) [14], and its reduction in SMA children (compared with healthy peers) should be the consequence of the differing FFM components. Only one study used Bioelectrical Impedance Analysis (BIA) [11], a validated method to measure body water [15], in SMA children, but it focused exclusively on the amount of FM, and did not provide data on the amount and distribution of body water across intra- and extra-cellular compartments. Furthermore, no previous study has investigated protein and mineral compartments. With regard to energy metabolism, only one study evaluated REE by indirect calorimetry in SMAII children [11], showing 18–21% reduction of the expected value, depending on the predictive equations used, while REE has never been explored in SMAI children.

The main purpose of this study was to assess total and regional fat, lean, mineral masses and body water (total, intra- and extra-cellular) in a sample of SMA type I children to compare their data with those of SMA type II children, matched for age and sex and adjusting for body size. This analysis aimed to show the regions and tissue compartments in which differences are found. The second purpose was to compare REE among SMA type I and II, also on the basis of body composition.

## 2 Materials and methods

### 2.1 Subjects and study design

Thirty SMA children was recruited, between April 2015 and May 2016, from 2 clinical SMA referral centers in Italy (Developmental Neurology Unit, Carlo Besta Neurological Institute Foundation, Milan, Italy and S.A.PRE., Early Habilitation Service, Mangiagalli e Regina Elena Hospital, Milan, Italy) both involved in a large ongoing study on nutritional status in SMA children. Including criteria for the enrollment were: genetically confirmed diagnosis of SMA types I or II, age 1–10 years, ability to lie on DEXA scanning table; had no tracheotomy or assisted ventilation for more than 16 h; there was no acute infection of any kind. The SMAII children were required to be the same sex, and to be within 6 months in age of the matched SMAI child. Each child underwent the following measurements and instrumental analysis on the same morning at the International Center for the Assessment of Nutritional Status (ICANS), University of Milan: anthropometric measurements [Body Weight (BW), Supine Length (SL), Arm Length (AL), Femur Length (FL), Tibia Length (TL)], DEXA [FM, lean body mass (LBM), and bone mineral content (BMC) total and segmental masses], BIA [Total Body Water (TBW), Intra Cellular Water (ICW), Extra Cellular Water (ECW)] and indirect calorimetry [REE, respiratory quotients (RQ)].

The Institution Review Board approved the study protocol, which complied with the Helsinki declaration tenets.

Before beginning the study the parents of the participating children gave their written informed consent.

### 2.2 Anthropometric measurements

All anthropometric measurements were collected by the same well-trained dietician by applying the conventional criteria and following recognized measuring procedures [16]. BW was measured by a wheelchair scale to the nearest 100 g, the subject and wheelchair were weighed together, then the wheelchair was weighed alone, the difference in the two measures gave the weight of the subject.

SL was measured by a non-elastic tape to the nearest 0.1 cm, on the child's right side. The child lay on its back on an appropriate exam table with the Frankfort plane perpendicular to the table (support), shoulders and buttocks resting against the table, arms along the trunk, palms facing up, legs as straight as possible and in contact with the table (board). In cases of scoliosis and contractures, segmental lengths were taken three times and the mean measurement recorded.

Body Mass Index (BMI) was calculated by the following formula: BW (Kg)/SL^2^ (m^2^). Sex-specific weight, length and BMI-Z-scores were derived using the 2000 Centers for Disease Control and Prevention (CDC) Growth Charts [17].

According to CDC guidelines a child with BMI-Z-scores value below the −2, between −2 and +2, between +2 and +3 or above +3 was considered underweight, normal weight, overweight or obese, respectively [17].

The measurements of AL, FL TL, all approximated to the nearest 0.1 cm, were taken along the child's right side by a non-elastic tape and with the child lying supine on the exam table, as for the SL measurement. For AL the right arm was extended laterally and bent at the elbow to make a 90° angle, palm facing down. The tape measure was then run down along the back of the arm from the end of the spine of the right scapula to the tip of the olecranon process. FL, measured along the anterior surface of the femur and with the right knee bent at a 90° angle, was taken from the inguinal crease, just below the iliac spine, to the proximal border of the patella. TL, measured with the right knee flexed, foot on the table, was taken from the knee joint (mid-patella) to the lower edge of the medial malleolus.

### 2.3 Dual-energy X-ray absorptiometry

DEXA scans (iDXA; General Electric, formerly Lunar Corp., Madison, WI) equipped with a pediatrics software application were used to obtain the body composition (BC) of the children. DEXA provides measurements of soft tissue and bone for the total body and the sub regions (arm, trunk, leg) including FM (g), LBM (g) and BMC (g). The fat free mass (FFM) was calculated by adding BMC to LBM. The fat mass percentage (FM%) was obtained as 100% × [total body fat mass(g)]/[total body mass (fat mass + lean mass + bone mass of total body) (g)]. Fat mass (FMI, kg/m^2^) and fat-free mass indexes (FFMI, kg/m^2^) were calculated by dividing FM and FFM by the squared height, respectively. To investigate the proportion between FFM and FM their ratio was also calculated. The total FM and BMC percentages of BW were interpreted according to the body composition of reference children [18], calculating the percentage of agreement between measured FM% and BMC% with the respective reference values for sex and age. Total body BMD was calculated as the amount of mineral matter per square centimeter of bone (g/cm^3^).

The scanning of the children was done with them lying supine on the table, their feet in a neutral position and arms resting along their sides, palms facing upwards. The DEXA scans, performed by well-trained and certified research staff, were all done using the one device and the same software (enCORE, 2010), for an average measuring time of 10 min. The exposure to radiation was <7 mSv. Daily quality-assurance was tested according to manufacturer directions. The DEXA scans were analyzed using a custom made software that allows BMC measurement in close relation to metal orthopedic implants, by exclusion of no-osseous pixels.

### 2.4 Bioelectrical impedance analysis

To measure the impedance a tetrapolar 8-point tactile electrode system (InBody S10, Biospace, Seoul, Korea) was used at 1, 5, 50, 250, 500 and 1000 kHz. Measurements were made of each child's trunk, right and left arms, and right and left legs. To estimate the total body impedance the segmental impedance values were added together. While child lay in comfort on a cot the dietitian applied the device's contact handles to the eight electrodes (2 on each hand and 2 on each foot). Manufacturer's predictive equations were applied to calculate TBW, ICW and ECW. Children with metallic implants were excluded from the measurements. The BIA coefficient of variation for intra-examination was 0.8%. TBW, ICW and ECW percentages of body weight values were interpreted according to the body composition of reference children [18] by calculation the percentage of agreement between measured TBW, ICW and ECW with those of the respective matching reference children for sex and age.

### 2.5 Resting energy expenditure

To measure oxygen consumption (VO_2_) and carbon dioxide production (VCO_2_) we used an open-circuit ventilated-hood system (Sensor Medics 29, Anaheim, CA, USA),. We took the measurements in a thermoneutral environment (ambient temperature 24–26 °C) devoid of external stimuli. At the beginning of each test the calorimeter was calibrated: there were two reference gas mixtures (26% O_2_ and 74% N_2_; 16% O_2_, 4.09% CO_2_ and 79.91% N_2_, respectively). Children were fasted for at least 6 h. Data collection time was at least 20 min, with a 5 min run-in time for stabilization and time to allow the children to get used to the canopy and instrument noise. Steady state was determined by five consecutive minutes in which VO_2_ and VCO_2_ variations were less than 10%. Children were not tested unless they had stable respiratory function for at least 1 h. Averaging the steady state values allowed the determination of 24 h REE, done by using the abbreviated Weir equation [19]: REE Kcal/day = (3.941 VO_2_ mL/min + 1.106 VCO2 mL/min) × 1.44. The ratio VCO_2_/VO_2_ gave the RQ. Measured REE values were compared with predicted REE values (pREE) based on the Harris-Benedict and the Schofield equations [20], [21].

### 2.6 Statistical analysis

All continuous variables are expressed as means ± standard deviations, while discrete variables are expressed as frequency and percentage. The mean values of each variable of interest were compared between the SMAI and SMAII children using the Test-T, while the distribution of the discrete variables was compared using Fisher's exact test. All analysis were performed by SPSS version 21.0 for Windows (SPSS, Inc., Chicago, IL, USA). A value of p < 0.05 was considered statistically significant.

## 3 Results

### 3.1 Sample

Thirty children (15 with SMAI, 15 with SMAII) were included in the analysis in accordance with the purposes of the study. As required by the study design, the two groups were similar for sex distribution (M/F = 9/6 vs 9/6) and age (3.6 ± 1.9 vs 3.5 ± 1.8 years, p = 0.99). Of the recruited children, 5 SMAI children had percutaneous endoscopic gastroscopy (PEG), while 9 SMAI and 1 SMAII children had nocturnal mechanical ventilation (8–12 h). At the time of enrollment the children were receiving the best possible supportive care, and were being followed according to the guidelines set out in the Consensus Statement for Standard of Care in SMA [22].

### 3.2 Anthropometrical measurements and nutritional status indexes

Anthropometrical measurements and nutritional status indexes are reported in Table 1.

**Table 1:** Anthropometrical measurements in SMAI and SMAII children.

	SMAI (n = 15)		SMAII (n = 15)		p Value
	Mean	sd	Mean	sd	
Age	3.6	1.9	3.5	1.8	0.990
**Anthropometric measurements**					
Body weight (kg)	12.1	3.4	13.1	2.6	0.367
Body weight (Z-score)	−2.7	1.6	−1.4	1.3	**0.018**
Supine length (cm)	100.0	16.1	96.6	11.7	0.509
Supine length (Z-score)	1.2	2.1	−0.1	1.7	0.082
Arm length (cm)	18.4	3.9	19.0	3.5	0.677
Femur length (cm)	21.8	4.4	22.2	4.7	0.813
Tibia length (cm)	18.2	4.4	17.0	2.9	0.404
**Nutritional status indexes**					
BMI (kg/m^2^)	12.1	2.2	14.1	1.7	**0.009**
BMI (Z-score)	−6.3	4.4	−2.1	2.3	**0.009**
Weight/length (kg/cm)	0.119	0.021	0.135	0.162	**0.030**

Abbreviations: BMI = Body Mass Index.

Statistically differences (p < 0.05) are reported in bold.

The groups had similar BW. However, children with SMAI had a lower weight-Z score compared to children with SMAII (p = 0.018). All the children with SMAI and 91.7% of those with SMAII had a BW below the median value of the reference group. Nonetheless, 75.0% and 41.7% of SMAI and SMAII children respectively had a BW Z-score <−2 (p = 0.214). SL was also similar in the two groups. SMAI children tended to have a higher score for supine length-Z (p = 0.082) than SMAII. Furthermore 66.7% children with SMAI and 40.0% of children with SMAII had a SL above the median value of the reference group. Moreover, 25.0% of children with SMAI and 8.3% of children with SMAII had a SL-Z score >2 (p = 0.295). The groups showed no differences for arm, femur and tibia length.

With regard to the nutritional status indexes, children with SMAI had a significantly lower BMI and BMI-Z score than children with SMAII. All children with SMAI and 83.3% of children with SMAII had a BMI below the median value of the reference group. In addition, a higher prevalence of children with a BMI-Z score <−2 was observed in SMAI than in SMAII (83.3% vs 41.7%, p = 0.045); on the contrary, none were overweight or obese in either group. Finally, weight for height index was significantly lower in SMAI.

### 3.3 Total and regional body composition measurements

**Fig. 1:**
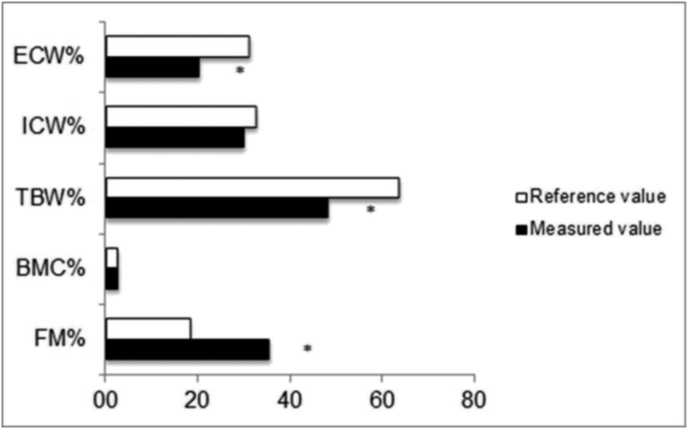
Comparison of body water, bone mineral content and total fat mass percentages between all SMA patients and children reference values.

Total FM, BMC, TBW, ICW and ECW percentage values of BW in the whole sample, compared with the BC values of the reference children are shown in Fig. 1.

**Fig. 2:**
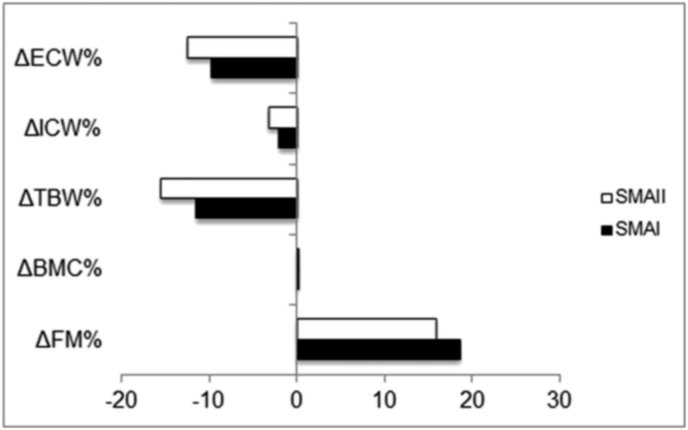
Comparison of body water, bone mineral content and total fat mass percentages between SMAI and SMAII patients.

As a group, all the children had a high FM percentage compared to the respective reference values for sex and age (mean percentage of overfat: +19,9 ± 8,0%, min + 2,5% max + 34%). Conversely, the BMC percentages did not differ. As required by the study design, children with metallic implants were excluded from the BIA. Therefore, hydration status was assessed on a sub-group of 20 children (10 SMAI and 10 SMAII). TBW and ECW were significantly lower than the reference values for sex and age (p < 001 and p = 0,01, respectively). The same results were found on analyzing SMAI and SMAII children separately, with no difference in the groups (Fig. 2).

Table 2 shows the body composition parameters.

**Table 2:** Total and regional fat, lean, mineral masses and body water in SMAI and SMAII children.

	SMAI (N = 15)		SMAII (N = 15)		p Value
	Mean	sd	Mean	Sd	
**Total and segmental fat mass**					
FM (%)	36.9	7.0	34.2	6.6	0.277
FM (g)	4502.3	1965.6	4553.8	1504.6	0.936
FM arms (g)	543.1	287.1	561.7	185.7	0.834
FM legs (g)	1763.9	827.1	1971.5	695.2	0.463
FM trunk (g)	1642.3	846.2	1451.6	665.8	0.498
FMI	4.4	1.4	4.9	1.3	0.354
**Total and segmental fat free mass**					
FFM (%)	63.1	7.0	64.7	7.6	0.547
FFM (g)	7290.0	1729.1	8410.1	1508.4	**0.039**
FFM arms (g)	461.8	187.5	587.3	165.8	0.063
FFM legs (g)	1285.7	499.7	2130.8	2212.9	0.160
FFM trunk (g)	3342.4	686.7	3969.3	803.0	**0.029**
FFMI	7.4	1.2	9.1	1.1	**<0.001**
**Total and segmental lean body mass**					
LBM (%)	60.4	6.9	61.9	7.5	0.564
LBM (g)	6971.8	1637.1	8041.7	1427.7	**0.037**
LBM arms (g)	443.9	180.7	562.0	158.0	0.067
LBM legs (g)	1254.3	488.1	2094.5	2205.2	0.161
LBM trunk (g)	3261.6	670.1	3870.7	789.9	**0.031**
**Total and segmental bone mineral content**					
BMC (%)	2.7	0.3	2.8	0.3	0.450
BMC (g)	318.2	97.5	368.3	92.6	0.160
BMC arms (g)	17.8	7.4	25.2	9.6	**0.025**
BMC legs (g)	31.4	12.7	36.3	12.4	0.295
BMC trunk (g)	80.7	28.8	98.6	31.3	0.115
BMD	0.477	0.089	0.535	0.077	0.067
**Body water**					
TBW (%)	52.3	14.3	48.2	10.3	0.475
TBW (l)	6.1	1.4	6.5	1.5	0.531
ICW (%)	30.6	8.9	29.4	8.3	0.751
ICW (l)	3.6	0.9	4.0	1.1	0.150
ECW (%)	21.7	5.5	18.9	2.2	0.146
ECW (l)	2.5	0.5	2.6	0.4	0.150

Abbreviations: FM = fat mass; FMI = Fat Mass Index; FFM = fat free mass; FFMI = Fat Free Mass Index; LBM = lean body mass; BMC = bone mineral content; BMD = bone mineral density; TBW = Total Body Water; ICW = Intra Cellular Water; ECW = Extra Cellular Water.

Statistically differences (p < 0.05) are reported in bold.

As shown in Table 2, total and segmental FM did not differ in the groups. Children with SMAI had significantly low FFM and LBM compared to children with SMAII. Specifically, children with SMAI presented a reduced FFM and LBM in the trunk and arms, although in these latter the difference in the groups was only marginally significant. Total BMC was similar between groups. However, children with SMAI showed less BMC in the arms and tended to have lower BMD than children with SMAII (p = 0.067). The groups showed no significant differences in TBW, ICW and ECW.

### 3.4 Resting energy expenditure

Table 3 shows the predicted and measured REE values.

**Table 3:** Gas exchange volumes and resting energy expenditure of children with SMAI and II.

	SMAI (N = 15)		SMAII (N = 15)		p Value
	Mean	Sd	Mean	sd	
**Resting energy expenditure (indirect calorimetry)**					
VO_2_	0.097	0.020	0.099	0.017	0.791
VCO_2_	0.082	0.020	0.087	0.021	0.522
RQ	0.839	0.057	0.880	0.157	0.355
REE (kcal/die)	684	143	703	122	0.707
REE/weight (kcal/kgBW)	58	10	54	7	0.215
REE/FFM (kcal/kgFFM)	95	12	84	11	**0.017**

Abbreviations: VO_2_ = Volume of O_2_; VCO_2_ = Volume of CO_2_; RQ = Respiratory Quotient; REE = Resting Energy Expenditure; BW = Body Weight; FFM = Fat Free Mass.

Statistically differences (p < 0.05) are reported in bold.

The volumes of O_2_ consumed and CO_2_ produced, respiratory quotient, REE (absolute value, Kcal/day) and REE for unit of BW were similar in the groups. Conversely, we observed a higher REE for units of FFM in children with SMAI compared to children with SMAII (p = 0.017).

**Fig. 3:**
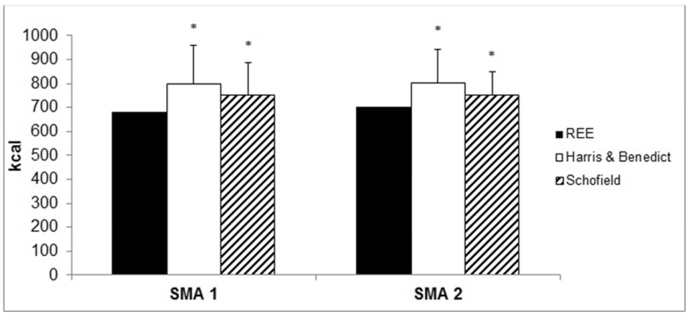
Comparison between measured values and predictive formulas of Resting Energy Expenditure.

In SMAI children, predictive formulas (Harris and Benedict, as well as Schofield) significantly overestimated REE (+13% and +11%, p = 0.014, p = 0.005, respectively) (Fig. 3). Similarly, in SMAII the predictive formulas significantly overestimated REE (+9% and +7%, p = 0.022, p = 0.021, respectively).

## 4 Discussion

Despite the established differences in SMAI and II neurological domains, the differences in BC and REE remain unclear. The present study is the first to investigate differences in total and regional fat, lean and mineral masses, body water, and REE in well matched SMAI and II children. The comparison of SMAI children with SMAII showed similar BW, SL and segmental lengths, significantly lower weight- and BMI-Z score, double severe malnutrition prevalence (BMI-Z score <−2), and a significantly lower BW/SL index. These data agree with previous studies [9], [10], [23], but need a more general consideration from the nutritional aspect. More than 50% of SMA children showed an above average SL-Z score, and none were stunted (chronic under-nutrition due to inadequate energy and protein intake). No SMA children had a FM% lower than the reference values [18]. SMAI children had total and regional fat masses and FMI, similar to SMAII, whereas total FFM, LBM and FFMI were lower in SMAI, particularly at the trunk level.

This seems more related to the magnitude of neurofunctional impairment, rather than to nutritional derangement. Indeed, SMAI had higher motor function impairment at both the axial level and in the proximal limbs: by definition they cannot sit unsupported, and proximal muscles are more affected by muscle weakness than distal, and the intercostal and axial muscle groups more than the diaphragm [24]. Our results suggest that SMA children, particularly SMAI, waste (BW- and BMI-Z scores), due to FM and FFM disproportion, not because of insufficient energy intake.

Misinterpretation leads to overfeeding, thus increased FM, worsening neurofunctional and respiratory conditions. A previous study [12] investigated FMI and FFMI in a cohort of 25 children and adolescents with SMA I, II and III; compared to reference data they had reduced FFMI and increased FMI; SMAI and II were not compared as only 2 SMAI patients were included in the cohort. Our SMAI and II study shows FFMI and FMI values similar to those of Sproule et al. [12], and a significant difference in FFMI, but not in FMI, indicating that body composition of SMAI and SMAII suggests overweight rather than underweight.

The major compartment of the human body is FFM, composed by water, protein, osseous and non-osseous minerals and some glycogen [14]. We measured body water by BIA, a valid method already applied to investigate hydration status in other pediatric disabilities [25], [26]. We also measured the mineral osseous component by DEXA (gold standard method). Regarding hydration, we found the TBW% and ECW% to be significantly lower in SMA children, compared to reference values, without significant SMAI and II differences. These data are partially consistent with the peculiar body composition of SMA. As expected, lower FFM resulted in lower TBW as FFM hydration [18]. As for TBW decrement, we expected to find lower ICW and ECW amounts. However, only ECW was lower than the reference, suggesting over-hydration of body cell mass. We have been unable to compare our results with other studies as this is the first study using BIA to investigate hydration in SMA. Thus caution is needed in interpreting the results. Our explorative results encourage BIA use in monitoring hydration status, and highlight the need to ascertain BIA applicability to SMA children by validation studies using deuterium oxide dilution techniques.

Concerning mineral osseous amount, BMC% values were similar to reference values [18]. Comparing SMA groups, the BMC in SMAI children was significantly reduced at arm level, whereas total BMD was only marginally lower. Studies on BMD in SMA patients are few [27], [28], and none investigated BMC. Recently, Vai et al. [13] found that BMD in young SMA II and III children was below the −1.5 Z score at spine level, with a high incidence of vertebral fractures, confirming high osteopenia and osteoporosis risk in young SMA subjects, with their well-known complications characterized by unloading and immobilization. We were unable to establish the degree of osteopenia and osteoporosis in our sample as BMD was assessed by total body scan; Z score reference values for the diagnosis of osteopenia and osteoporosis are only available for specific osseous segments. Our data are in agreement with that of Vai et al. as SMAI children have, by definition, upper and lower motor impairment, SMA II children being much less compromised in upper limb function, confirming the correlation of BMC with motor deficit severity.

FFM is the active metabolic compartment with strong energy metabolism correlation. This was why we compared REE in SMAI and II, based on BC. Absolute REE did not differ, nor did the REE value normalized for BW. On normalizing it for FFM, we found that SMAI had higher basal energy expenditure than SMAII. This suggests more expensive muscle work for SMAI, probably because of high respiratory function impairment. On comparing REE with the values obtained by predictive equations, we found that the predictive equations overestimated caloric consumption. In a third of SMAI and in a quarter of SMAII the predictive equations overestimated REE more than 20%. Only one previous study assessed REE in SMAII patients [29], the authors reporting a higher deviation from real energy consumption by predictive equation, differing from our results possibly because of the different age range investigated. Patients recruited by Cutillo et al. [29] were twice the median age of ours (6.3 vs 3.5 years). We can hypothesize that older children could have a worse BC, leading to greater REE changes. According to Cutillo et al., we found the mean RQ in line with reference values [30] without differences between SMAI and II. The disaccord between measured and predicted REE reflect the nutritional management of SMA children, particularly in SMAI. Excessive food intake, consequent to overestimation of energy needs, particularly in children. A strength of the study is the data quality: SMAI and SMAII patients had similar sex distribution and age; the same dietician performed anthropometric measurements and indirect calorimetry; we measured BC and REE with gold standard methods.

Potential limitations to be addressed: a restricted number of children were enrolled, although our population appears homogeneous in relation to the supportive care operated according to the guidelines of the Consensus Statement for SMA. Larger studies involving different age ranges, and neurofunctional status, are needed to confirm our results. We studied hydration status by BIA, which provides an indirect measurement of TBW, ICW and ECW by measuring reactance and resistance. Compared to the deuterium dilution technique, the gold standard methods to measure body water, BIA is a valid method, but validation studies are needed to produce a specific predictive equation for SMA to ensure interpretability of BIA results in SMA children. Furthermore, as this is a cross-sectional study no understanding is possible of whether nutritional status impairment is the cause, or the consequence, of the changes in body composition and REE reduction. To define the time course of body composition and REE changes in SMA children, longitudinal studies are need.

This study has shown that SMAI children, compared to SMAII, had i) similar total and regional FM and FMI, ii) lower total FFM and LBM, specifically at trunk and arm levels, and FFMI, iii) lower BMC at arm level and iv) similar hydration status. Taken together, the data suggest that BW and BMI Z-score measurements may be misleading in assessing nutritional status, particularly in SMAI, and could lead to erroneous undernutrition diagnoses, and, in turn, to overfeeding dietary intervention. Nevertheless, REE per FFM unit is higher in SMAI, suggesting hypermetabolism of a quantitatively reduced FFM. Thus, the energy requirements of SMAI and SMAII children could differ in relation to their BC. Based on these results, we recommend direct BC and REE measurements in the nutritional care process until SMA-specific predictive equations become available.

## Sources of funding

Support for this study was given by the Italian Association of SMA Families (Famiglie SMA, 2015–2016 contribution) and by Fondazione Telethon (Application GUP15014, 2015, Italy).

## Authorship

The research was designed by SB and AB and conducted by SB: who also performed the statistical analysis and wrote the manuscript; RD: collected all the anthropometric measurements; CM and GB collected all neurofunctional parameters and enrolled the sample subjects; EG and GP helped with the data interpretation; AL performed statistical analysis; The primary responsibility for final content was in the hands of SB: The final manuscript was read and approved by all the authors.

## Conflict of interest

There was no financial conflict of interest on the part of any of the authors.
